# Multimorbidity transitions and the associated healthcare cost among the Finnish adult population during a two-year follow-up

**DOI:** 10.1177/26335565231202325

**Published:** 2023-09-12

**Authors:** Katja Wikström, Miika Linna, Eeva Reissell, Tiina Laatikainen

**Affiliations:** 1 Institute of Public Health and Clinical Nutrition, 163043 University of Eastern Finland, Kuopio, Finland; 2 Department of Public Health and Welfare, 3837 Finnish Institute for Health and Welfare, Helsinki, Finland; 3 Department of Health and Social Management, 163043 University of Eastern Finland, Kuopio, Finland; 4 Institute of Healthcare Engineering, Management and Architecture, 174277 Aalto University, Helsinki, Finland; 5 Joint Municipal Authority for North Karelia Social and Health Services, Joensuu, Finland

**Keywords:** Multimorbidity, prevalence, incidence, cost, healthcare, sustainability

## Abstract

**Background:**

Ageing of the population increases the prevalence and coexistence of many chronic diseases; a condition called multimorbidity. In Finland, information on the significance of multimorbidity and its relation to the sustainability of healthcare is scarce.

**Aim:**

To assess the prevalence of multimorbidity, the transitions between patient groups with and without multiple diseases and the associated healthcare cost in Finland in 2017–2019.

**Methods:**

A register-based cohort study covering all adults (*n* = 3,326,467) who used Finnish primary or specialised healthcare services in 2017. At baseline, patients were classified as ‘non-multimorbid’, ‘multimorbid’ or ‘multimorbid at risk’ based on the recordings of a diagnosis of interest. The costs were calculated using the care-related patient grouping and national standard rates. Transition plots were drawn to observe the transition of patients and costs between groups during the two-year follow-up.

**Results:**

At baseline, 62% of patients were non-multimorbid, 23% multimorbid and 15% multimorbid at risk. In two years, the proportion of multimorbid patients increased, especially those at risk. Within the multimorbid at-risk group, total healthcare costs were greatest (€5,027 million), accounting for 62% of the total healthcare cost of the overall patient cohort in 2019. Musculoskeletal diseases, cardiometabolic diseases and tumours were the most common and expensive chronic diseases contributing to the onset of multimorbidity.

**Conclusion:**

Multimorbidity is causing a heavy burden on Finnish healthcare. The estimates of its effect on healthcare usage and costs should be used to guide healthcare planning.

## Introduction

Ageing of the population increases the prevalence and coexistence of many chronic diseases; a condition called multimorbidity. Globally, wide disparities in the prevalence estimates (ranging from 13%–72% in the general population) exist due to varying multimorbidity definitions and methodologies.^1,2^ According to the WHO definition, ‘multimorbidity is the coexistence of two or more chronic conditions in the same individual’, and it is the most used definition in scientific literature.^3^ However, some definitions also include acute conditions or use the cut-off point of three or more. The number of medical conditions included in the measures of multimorbidity has been shown to vary widely, ranging from a few conditions to hundreds.^4^ Also, there are differences in the methods to define multimorbidity, starting from the simple counting of diseases to more complex clustering and in data sources (i.e. register data and self-reports) between the previous studies.^5^ Due to these differences, it has been difficult to compare findings across populations and to develop solutions to tackle this growing public health problem which challenges western countries and the sustainability of their social and healthcare systems.

Previous research has shown that patients with prevalent multimorbidity are often older and have increased risk factor levels, high use of health services, more hospitalisations and higher mortality compared with healthy populations or patients with only one chronic condition.^6^ Further, higher rates of primary and specialised care visits, medication use, emergency department visits and hospital admissions in patients with multiple chronic conditions have all been connected to rising healthcare expenses.^7^ With the increasing prevalence of multimorbidity, healthcare expenses and resource consumption rise dramatically, even exponentially. The National Institute for Health and Care Excellence (NICE) has published a guideline on assessing and managing patients with multimorbidity.^8^ In accordance with the NICE Guidelines, the Finnish Medical Society Duodecim has published the Current Care Guidelines, which aim to improve the care of people with multimorbidity and is intended as a basis for treatment decisions.^9^ These guidelines indicate the situations in which the identification of patients with multiple diseases and the individual planning of treatment are essential. Such situations include patients with both physical illnesses and mental health problems, patients with frailty, patients who use many health services, patients with multiple medications and patients who have difficulties managing their everyday lives. These multimorbid patients are at higher risk of premature death, hospitalisation and increased costs than others.^8,9^

Managing multimorbidity is difficult for patients and healthcare systems, and multimorbid patients frequently require primary and secondary care services. Existing healthcare services are not adequately designed to meet the challenges of multimorbidity; they have often been focused on the care of single diseases.^10^ Social and healthcare systems that can manage the emerging issues associated with multimorbidity and related costs are urgently needed. Country-specific knowledge about the prevalence of multimorbidity and estimates of its effect on healthcare utilisation and costs are required to guide healthcare planning and improve care quality. In Finland, information on the significance of multimorbidity and its relation to the sustainability of healthcare is scarce. Therefore, this study aims to assess the prevalence of multimorbidity, the transitions between patient groups with and without multiple diseases, and associated costs in Finnish healthcare in 2017–2019.

## Data and methods

The data were extracted from the Finnish Care Registers (Care Register for Health Care and Register of Primary Health Care Visits) from 2015–2019. The registers provide information on a patient’s gender, age, and healthcare contacts. Each individual contact with a healthcare professional is recorded in the register, including information about the type of contact and diagnoses (ICD and ICPC codes). The study cohort includes all adults aged 18 years or older who used Finnish primary or specialised healthcare services in 2017. Altogether, 3,326,467 patients were included in the analyses.

### Classification of chronic diseases

Calderón-Larrañaga et al.^11^ provided a list of chronic conditions using ICD-10 codes and grouped all four-digit level codes into broader categories to measure multimorbidity in the older population. We modified the existing disease list using three-digit ICD-10 codes and added the corresponding ICPC-2 codes. In addition, we excluded the three-digit codes which included certain sub-codes for an acute version of the condition. Based on the codes, we identified all diagnoses of interest from the Care Registers and created broader disease groups.

Previously, Fränti et al. constructed a multimorbidity network covering all Finnish primary and specialised care services patient visits in 2015–2018 and related diagnoses represented as blocks of ICD-10 codes.^12,13^ Using the network data, we ensured that relevant diseases were comprehensively identified and grouped to assess multimorbidity. Furthermore, a multimorbidity measure was created based on these 34 disease groups and used for the analysis to understand the impact of multimorbidity on healthcare utilisation and costs in Finland. Supplementary Table 1 presents the disease groups and codes used to identify chronic diseases.

### Multimorbidity status

Multimorbidity status (non-multimorbid, multimorbid or multimorbid at risk) at baseline was determined based on the recordings of a diagnosis of interest using Finnish Care Register data. For the analysis, patients who were free from all selected diseases or had only one disease (i.e. disease or diseases from one disease group) were defined as ‘non-multimorbid’ at baseline. Patients with co-occurrence of two or more chronic diseases (i.e. diseases from ≥ 2 disease groups) were defined as ‘multimorbid’. ‘Multimorbid patients at high risk’ were patients with both physical illnesses and mental health problems, multimorbid patients with age-related physical weakness or disabilities (ICD-10 code R54; ICPC-2 codes: A28, N28, P28, Z28), or multimorbid patients who used emergency care services three times or more per year. In this study, the concept of “multimorbid patient at risk” was adopted from the guidelines.^8,9^ In the guidelines, the definition of multimorbid patients at risk includes a slightly broader set of criteria than we were able to use due to the limited availability of information from the register.

The non-multimorbid, multimorbid and multimorbid at risk patients were defined at the moment of their first visit to the health services in 2017. Only those who had at least some kind of a contact to health services in 2017 were included in the analyses. Data from prior years was used to capture the actual multimorbidity status at baseline. Those who had previously (between the years 2015-2017) or at that visit diagnoses of two or more chronic diseases were regarded as multimorbid. Similarly, those who fulfilled the criteria of being at risk by the first visit in 2017 were regarded as multimorbid at risk patients. Follow-up of each individual continued until death from any cause or the end of the two-year follow-up period. The date of death was received from the National Register of Causes of Death.

### Statistical analysis

Frequencies and percentages were used to describe the baseline characteristics in patient groups with and without multimorbidity and diseases contributing to the onset of multimorbidity and related costs in this patient cohort. The costs were calculated also in patient quartiles based on healthcare costs. The counting of costs started on the date of the first visit in 2017 and costs of all contacts were counted for 730 days (2 years) after the first visit in 2017. The costs were calculated using the care-related patient grouping and national standard rates. The costs for hospitalisations and outpatient hospital visits were based on the NordDRG patient grouping definitions, which use the International Classification of Diseases 10th revision (ICD-10) codes and the Finnish version of the Nordic Classification of Surgical Procedures (NCSP) codes for diagnostic and treatment procedures. The cost weights for DRG-grouped hospital discharges and outpatient hospital visits are calculated using large national samples of individual-level cost accounting data from Finnish hospitals by the national authorities. The unit cost estimates for community care bed days were derived from the national price list for unit costs of healthcare services in Finland and used with the individually calculated length-of-stay data. The primary care contacts were grouped according to the contact type (visit, call, home care, e-messages) and the healthcare professional. Each primary care contact type was priced according to the national price list for unit costs.^14^ Prices were adjusted to the 2017 level using the healthcare price index by Statistics Finland.

Transition plots were drawn to observe the transition of patients and costs between non-multimorbid, multimorbid, multimorbid at-risk and dead categories during the two-year follow-up.

## Results

The study cohort included 3,326,467 patients (1,431,025 men and 1,895,442 women) who had used public healthcare services in 2017. Of the patients, 62% (2,066,457) were non-multimorbid, 23% (771,690) multimorbid and 15% (488,320) multimorbid at risk at baseline (Table 1). The proportion of men was 45%, 42% and 39% in the non-multimorbid, multimorbid and multimorbid at risk group, respectively. At baseline, men and women without multiple diseases were more often younger (≤ 64 yrs) than multimorbid patients. In 2017, the total healthcare cost was €8,151 million for this patient population, and the healthcare cost per patient depended on the severity of multimorbidity; the total healthcare costs were the lowest among non-multimorbid patients (1,383€ per patient in 2017) and the highest among multimorbid patients at risk, i.e. among those with both physical illnesses and mental health problems, or with age-related physical debility or disabilities or who used emergency care services frequently (6,678€ per patient in 2017). The highest patient quartile incurred 80-90% of the total healthcare costs in each patient group.

**Table 1.** Baseline characteristics in patient groups with and without multimorbidity.

	2017	2017	2017
	Non-multimorbid	Multimorbid	Multimorbid at risk
Patients, n (%)	2,066,457 (62)	771,690 (23)	488,320 (15)
Men, n (%)	919,446 (45)	322,458 (42)	189,121 (39)
Age ≤ 64 yrs, n (%)	1,647,656 (80)	333,855 (43)	213,684 (44)
Total healthcare costs, € million	2,819	2,403	2,929
Total costs per patient, €	1,383	3,217	6,678
Cost in patient quartiles, € million (% of costs in the patient group)			
I	0	22 (1%)	17 (1%)
II	61 (2%)	109 (5%)	116 (4%)
III	248 (9%)	298 (12%)	390 (13%)
IV	2,510 (89%)	1,970 (82%)	2,410 (82%)

### Multimorbidity transitions and associated healthcare costs during the two-year follow-up

**Figure 1.**
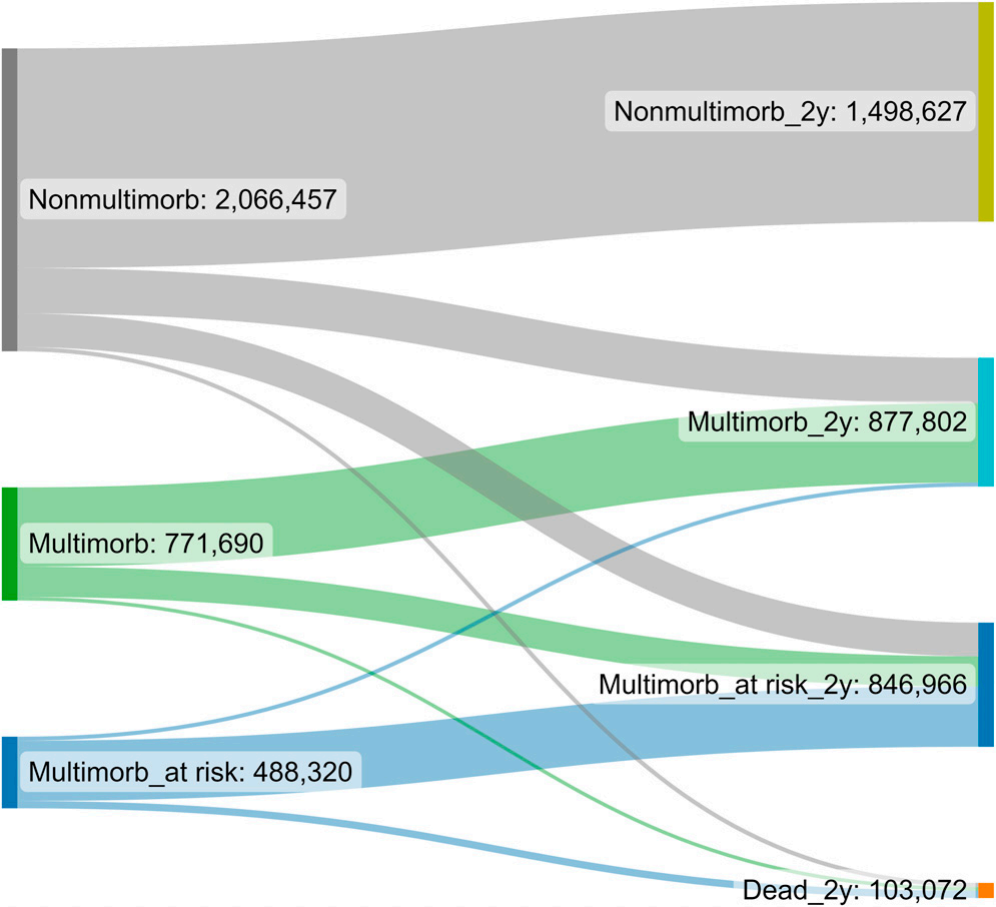
Transitions of patients (n) with and without multimorbidity in the 2017 patient cohort during the two-year follow-up.

We found a considerable number of patients moving from the non-multimorbid group to multimorbid and multimorbid at-risk groups during the two-year follow-up (Figure 1). After the follow-up, altogether, 45% (1,498,627) of patients were non-multimorbid, 26% (877,802) were multimorbid, and 25% (846,966) were multimorbid at risk of poor outcomes, and 3% (103,072) had died. During the follow-up, the observed transitions between patient groups with and without multimorbidity were similar among men and women. However, multimorbidity was more prevalent among women than among men. In 2019, 60% of multimorbid patients were women, and 40% were men (data not shown).

**Figure 2.**
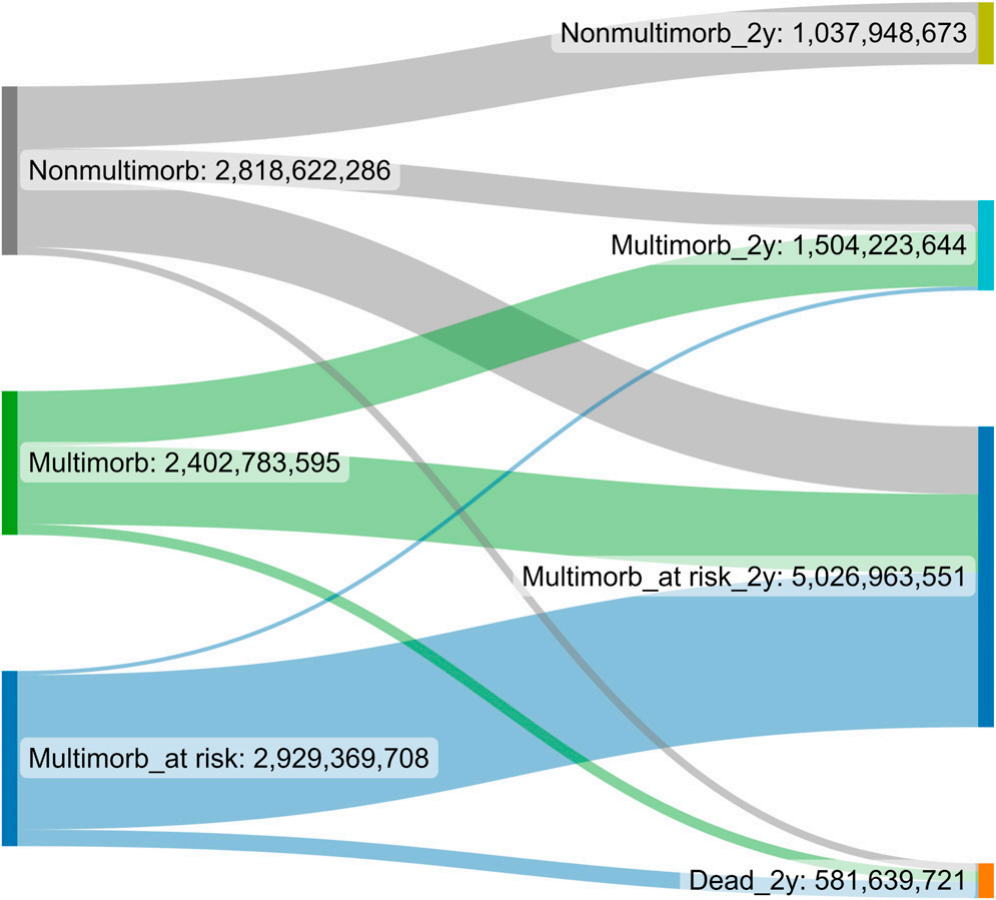
Costs (€) associated with multimorbidity transitions in the 2017 patient cohort during the two-year follow-up.

In this prevalent patient cohort, the proportion of multimorbid patients at risk increased the most during the follow-up (15% in 2017 vs 25% in 2019). It was also the costliest patient group, costing €5,027 million and accounting for 62% of the total healthcare cost of this patient cohort in 2019 (Figure 2). Correspondingly, 18% (€1,504 million) of the total healthcare cost was associated with healthcare usage among multimorbid patients and 13% (€1,038 million) among non-multimorbid patients in 2019. In 2017, the proportions of healthcare cost were 35% (€2,819 million), 29% (€2,403 million) and 36% (€2,929 million) in the non-multimorbid, multimorbid, and multimorbid at-risk groups, respectively.

### Incidence of multimorbidity among non-multimorbid patients during a 2-year follow-up in the 2017 patient cohort

Based on the absolute number of multimorbid patients in each disease group after the two-year follow-up, the ten most common patient groups becoming multimorbid were patients with back diseases (n = 47,000), other musculoskeletal diseases (n = 32,578), diabetes (n=23,285), hypertensive diseases (n = 22,473), chronic eye diseases, including blindness (n = 17,473), other diseases of the heart and pulmonary circulation (n = 16,821), cancer and in-situ carcinomas (n = 16,738), arthrosis (n = 14,696), mood disorders (n = 13,436), and neurotic, stress-related and somatoform diseases (n = 13,390) (Table 2).

**Table 2.** The most common patient groups becoming multimorbid and related healthcare costs during the two-year follow-up in the 2017 patient cohort.

Disease group	Total number of non-multimorbid patients having a diagnosis in 2017	Patients becoming multimorbid in the disease group during follow-up (n [%])	Cumulative 2-year healthcare costs in the group, million €
Back diseases	109,452	47,000 (43)	107
Other musculoskeletal diseases	72,161	32,578 (45)	75
Diabetes	43,776	23,285 (53)	80
Hypertensive diseases	39,805	22,473 (56)	66
Chronic eye diseases and blindness	38,189	17,473 (46)	63
Other diseases of the heart and pulmonary circulation	32,126	16,821 (52)	70
Cancer and in-situ carcinomas	34,733	16,738 (48)	79
Arthrosis	27,228	14,696 (54)	45
Mood disorders	27,488	13,436 (49)	50
Neurotic, stress-related and somatoform diseases	30,747	13,390 (44)	36

Out of the patients with hypertensive diseases at the baseline, 56% (22,473/39,805) became multimorbid, followed by the proportions of the patients with arthrosis (54%; 14,696/27,228), diabetes (53%; 23,285/43,776), and other diseases of the heart and pulmonary circulation (52%; 16,821/32,126). Of patients with back diseases at the baseline, 43% (47,000/109,452) became multimorbid, causing the highest cumulative healthcare costs (€107 million) during the two-year period. The second highest cumulative two-year healthcare costs were €80 million in the group of patients with diabetes, and the third highest cumulative costs were €79 million in the patient group with cancer and in-situ carcinomas (Table 2).

In addition, back diseases, hypertensive diseases, and cancers and in-situ carcinomas were the most common and costly ‘secondary diseases’ resulting in multimorbidity in this patient cohort in two years (Table 3). The cumulative two-year healthcare costs were the highest (€148 million) in patients becoming multimorbid due to back diseases (n = 63,983). Correspondingly, the cumulative costs were €122 million both in patients becoming multimorbid due to hypertensive diseases (n = 39,516) and cancers and in-situ carcinomas (n = 20,556) (Table 3). In the patient group that became multimorbid due to other diseases of the heart and pulmonary circulation, the two-year follow-up resulted in cumulative healthcare expenses of €109 million.

**Table 3.** The most common and expensive disease groups resulting in multimorbidity among non-multimorbid patients during the two-year follow-up in the 2017 patient cohort.

Disease group	The number patients becoming multimorbid due to the disease during follow-up	Cumulative 2-year healthcare costs, million €
Back diseases	63,983	148
Hypertensive diseases	39,516	122
Cancer and in-situ carcinomas	20,556	122
Other diseases of the heart and pulmonary circulation	24,193	109
Other musculoskeletal diseases	46,104	105
Diabetes	26,660	89
Mood disorders	21,930	83
Chronic eye diseases and blindness	21,863	80
Neurotic, stress-related and somatoform diseases	23,140	66
Arthrosis	19,906	62

Overall, different musculoskeletal diseases, cardiometabolic diseases, and tumours were the most common chronic diseases among non-multimorbid patients in 2017, contributing to the onset of multimorbidity and causing huge expenses during the two-year follow-up.

## Discussion

### Summary of findings

This study is one of the first to estimate the significance of multimorbidity and its relation to Finnish healthcare sustainability using the register data covering all adults who used primary or specialised healthcare services in Finland in 2017. This study provides information on the prevalence of multimorbidity in Finnish healthcare, the transitions between patient groups with and without multiple diseases and associated healthcare costs during the two-year follow-up.

At baseline almost two-thirds of the patients were non-multimorbid, 23% were multimorbid, and 15% were multimorbid at risk. During the two-year follow-up, a considerable number of patients moved from the non-multimorbid group to multimorbid and multimorbid at-risk groups. The proportion of multimorbid patients at risk increased the most; from 15% to 25% in two years. They had also the greatest total healthcare costs (€5,027 million), accounting for 62% of the patient cohort’s overall healthcare spending in 2019. During the follow-up, the most common chronic diseases contributing to the onset of multimorbidity and causing huge healthcare expenses were musculoskeletal and cardiometabolic diseases and tumours.

### Findings in context

Marked variations have been shown to exist among multimorbidity studies in terms of methodology and findings.^15,16^ Despite the variability, the results from the studies evaluating the effect of multimorbidity on healthcare utilisation and cost have shown to be relatively consistent. The review of Lehnert et al. included 35 international studies. It indicated a positive association between multimorbidity and healthcare use outcomes (physician visits, hospitalisations, use of medications) and healthcare cost outcomes (medication, out-of-pocket, total healthcare expenditures),^17^ and later the review of Wang et al. confirmed their findings on the economic burden of multimorbidity.^18^ Also, a systematic review of the UK literature showed that multimorbidity was associated with increased primary and specialised healthcare use and related costs (total costs, hospital costs, primary and dental care use, emergency department use, and hospitalisations).^19^ In addition, it has been shown that healthcare use and costs tend to increase with the number of chronic conditions.^7,17,20^ Several studies have also demonstrated that particularly multimorbidity with mental health disorders or frailty is associated with higher levels of health service use and a higher cost.^19, 21–23^

A systematic review by Kudesia et al. determined the incidence of multimorbidity and the order in which chronic conditions accumulate to result in multimorbidity.^24^ Of the 36 included studies, five analysed the accumulation of chronic conditions. In accordance with our findings, hypertensive and heart diseases and diabetes were the most common starting conditions resulting in later multimorbidity. Cerebrovascular diseases, mental health illnesses, chronic pain, osteoarthritis, cancer and hypercholesterolemia were reported as second or third conditions resulting in multimorbidity. Also, back pain is associated with an increased likelihood of comorbidity in previous studies.^25, 26^ However, starting conditions contributing to multimorbidity may vary by age. The study of Ashworth et al. found that coronary heart disease and diabetes were more common as starting conditions in those over 65, while depression was more common in those under 65 years old.^27^ All ages are affected by low back pain, which is a major leading contributor to the burden of disease.^28^

Recently, Tran et al. conducted a meta-analysis of 15 multimorbidity studies aiming to explore the cost of specific, most frequently reported, disease combinations in U.S. healthcare.^29^ Based on the results, there are differences in costs for treating different disease combinations. The least expensive to manage were disease combinations involving hypertension, and the most expensive were those with cancer and mental health conditions. However, the cost difference for treating these combinations may vary significantly between countries because of the different healthcare systems. Stokes et al. aimed to examine the potential of defining multimorbidity clusters impacting secondary care costs in England.^30^ They found that all combinations containing chronic kidney disease and hypertension, or diabetes and hypertension, made up a significant proportion of total secondary care costs. Similarly, Fränti et al. performed a cluster analysis of diagnoses using data from 58 million patient visits in Finnish primary and secondary healthcare.^12^ Based on their results, the total costs were the highest (€2.3 billion) in the cluster, including cardiovascular and metabolic problems. In our study, musculoskeletal diseases, cardiovascular and metabolic diseases and tumours were the costliest diseases contributing to multimorbidity in Finnish healthcare.

### Strengths and limitations of this study

The main strength of this study is the register data, including all public primary and secondary healthcare visits of the Finnish adult population from 2015–2019. The Finnish Care Registers (Care Register for Health Care and Register of Primary Health Care Visits) include the ICD and ICPC-2 codes for diagnosis. Healthcare professionals record the codes in the register. The completeness and accuracy of the Care Register for Health Care providing data on both primary- and secondary-level inpatient care have been previously documented and shown to vary from satisfactory to very good.^31^ The Register of Primary Health Care Visits provides additional data on outpatient primary healthcare activities. Recording activity in the register has shown to be at an acceptable level to be used for research purposes.^32^ However, the registers do not include data from private health services and may have left out information on patients who prefer and can afford private health services and privately provided occupational health services. These services total about €2 billion in costs, while the costs of specialised care are about €8 billion per year, and in primary care, about €3.5 billion.^33^

In addition, a comparison of the results of this study with the previous ones is challenging due to differences in data sources, methodologies, health systems and regions. Therefore, it is essential to provide country- and system-specific information on the impact of multimorbidity on healthcare utilisation and costs to inform policy and practice.^7^

### Implications for future research and clinical practice

The Finnish population is ageing, and the need for care is increasing. In Finland, the ongoing health and social services reform aims to centralise organisational structures to improve access to primary care and better integrate services.^34,35^ The ongoing reform offers possibilities to take action towards a more sustainable healthcare system, respond to the changes in care demand among the ageing population, and improve the continuity of care. This study shows how multimorbidity, especially multimorbid patients at risk, is causing a heavy burden and costs on Finnish healthcare. Furthermore, there seems to be reasonably small proportion of patients in all patient groups that cause most of the healthcare cost in that group. We would need better understanding about this phenomenon and whether service needs can be reduced among these patients. These timely results should be used to guide health service planning and targeting of preventative interventions and to deliver a good quality of care to patients with multimorbidity. Particular attention should be paid to patients with hypertensive or musculoskeletal diseases, which seem to be common conditions contributing to the onset of multimorbidity and are both conditions with prevention potential.

## Supplemental Material

Supplemental Material - Multimorbidity transitions and the associated healthcare cost among the Finnish adult population during a two-year follow-up Click here for additional data file. Supplemental Material for Multimorbidity transitions and the associated healthcare cost among the Finnish adult population during a two-year follow-up by Katja Wikström, Miika Linna, Eeva Reissell and Tiina Laatikainen in Journal of Multimorbidity and Comorbidity

## Data Availability

The data that support the findings of this study are available from the Finnish Institute for Health and Welfare registers, but restrictions apply to the availability of these data, which were used under license for the current study, and so are not publicly available due to Finnish data protection legislation. According to the legislation, register authorities give permissions to use register data including sensitive individual information (e.g., health data) to study specified research questions to named individuals who have signed a pledge of secrecy and they are not permitted to forward it to other researchers. Other researchers can apply for the data from the Health and Social Data Permit Authority Findata. Findata handles the data permit applications concerning Finnish Institute for Health and Welfare registers.^36^ https://www.findata.fi/en/services/data-requests/

## References

[1] The L . Making more of multimorbidity: an emerging priority. Lancet 2018;391(10131):1637. 29726322 10.1016/S0140-6736(18)30941-3

[2] Xu X Mishra GD Jones M . Evidence on multimorbidity from definition to intervention: An overview of systematic reviews. Ageing Res Rev 2017;37:53–68. 28511964 10.1016/j.arr.2017.05.003

[3] Multimorbidity : Technical Series on Safer Primary Care. Geneva: World Health Organization; 2016. Licence: CC BY-NC-SA 3.0 IGO.

[4] Ho IS Azcoaga-Lorenzo A Akbari A , et al. Examining variation in the measurement of multimorbidity in research: a systematic review of 566 studies. Lancet Public Health 2021;6(8):e587–e597. 34166630 10.1016/S2468-2667(21)00107-9

[5] Stirland LE González-Saavedra L Mullin DS , et al. Measuring multimorbidity beyond counting diseases: systematic review of community and population studies and guide to index choice. BMJ 2020;368:m160. 32071114 10.1136/bmj.m160 PMC7190061

[6] Marengoni A Angleman S Melis R , et al. Aging with multimorbidity: a systematic review of the literature. Ageing Res Rev 2011;10(4):430–439. 21402176 10.1016/j.arr.2011.03.003

[7] McPhail SM . Multimorbidity in chronic disease: impact on health care resources and costs. Risk management and healthcare policy 2016;9:143–156. 27462182 10.2147/RMHP.S97248 PMC4939994

[8] National Institute for Health and Care Excellence . (2016). Multimorbidity: clinical assessment and management [NICE guideline 56]. www.nice.org.uk/guidance/ng56 (2022-09-15).

[9] Multimorbid patient. Current Care Guidelines. Working group set up by the Finnish Medical Society Duodecim and The Finnish Association for General Practice. Helsinki: The Finnish Medical Society Duodecim, 2021. www.kaypahoito.fi (2022-09-15).

[10] Wallace E Salisbury C Guthrie B , et al. Managing patients with multimorbidity in primary care. BMJ 2015;350:h176. 25646760 10.1136/bmj.h176

[11] Calderón-Larrañaga A Vetrano DL Onder G , et al. Assessing and Measuring Chronic Multimorbidity in the Older Population: A Proposal for Its Operationalization. J Gerontol A Biol Sci Med Sci 2017;72(10):1417–1423. 28003375 10.1093/gerona/glw233 PMC5861938

[12] Fränti P Sieranoja S Wikström K , et al. Clustering Diagnoses From 58 Million Patient Visits in Finland Between 2015 and 2018. JMIR Med Inform 2022;10(5):e35422. 35507390 10.2196/35422 PMC9118010

[13] Multimorbidity network analysis. University of Eastern Finland. http://cs.uef.fi/ml/impro/DiagnosisClusters/ (2022-12-02).

[14] Mäklin S Kokko P . Terveyden- ja sosiaalihuollon yksikkökustannukset Suomessa vuonna 2017. Työpaperi 21/2020. Terveyden ja hyvinvoinnin laitos.

[15] Fortin M Stewart M Poitras ME , et al. A systematic review of prevalence studies on multimorbidity: toward a more uniform methodology Ann Fam Med. 2012;10(2):142–151. 22412006 10.1370/afm.1337 PMC3315131

[16] Violan C Foguet-Boreu Q Flores-Mateo G , et al. Prevalence, determinants and patterns of multimorbidity in primary care: a systematic review of observational studies. PLoS One 2014;9(7):e102149. 25048354 10.1371/journal.pone.0102149 PMC4105594

[17] Lehnert T Heider D Leicht H , et al. Review: health care utilization and costs of elderly persons with multiple chronic conditions. Med Care Res Rev 2011;68(4):387–420. 21813576 10.1177/1077558711399580

[18] Wang L Si L Cocker F , et al. A Systematic Review of Cost-of-Illness Studies of Multimorbidity. Appl Health Econ Health Policy 2018;16(1):15–29. 28856585 10.1007/s40258-017-0346-6

[19] Soley-Bori M Ashworth M Bisquera A , et al. Impact of multimorbidity on healthcare costs and utilisation: a systematic review of the UK literature. Br J Gen Pract 2020;71(702):e39–e46. 33257463 10.3399/bjgp20X713897 PMC7716874

[20] Onder G Palmer K Navickas R , et al. Time to face the challenge of multimorbidity. A European perspective from the joint action on chronic diseases and promoting healthy ageing across the life cycle (JA-CHRODIS). Eur J Intern Med 2015;26(3):157–159. 25797840 10.1016/j.ejim.2015.02.020

[21] Calderón-Larrañaga A Abad-Díez JM Gimeno-Feliu L , et al. Global health care use by patients with type-2 diabetes: Does the type of comorbidity matter? Eur J Intern Med. 2015;26(3):203–210. 25765442 10.1016/j.ejim.2015.02.011

[22] Vetrano DL Palmer K Marengoni A , et al. Frailty and Multimorbidity: A Systematic Review and Meta-analysis. J Gerontol A Biol Sci Med Sci 2019;74(5):659–666. 29726918 10.1093/gerona/gly110

[23] Zhao Y Zhang P Oldenburg B , et al. The impact of mental and physical multimorbidity on healthcare utilization and health spending in China: A nationwide longitudinal population-based study. Int J Geriatr Psychiatry 2021;36(4):500–510. 33037674 10.1002/gps.5445

[24] Kudesia P Salimarouny B Stanley M , et al. The incidence of multimorbidity and patterns in accumulation of chronic conditions: A systematic review. Journal of Multimorbidity and Comorbidity 2021;11:26335565211032880. 34350127 10.1177/26335565211032880 PMC8287424

[25] Hestbaek L Leboeuf-Yde C Manniche C . Is low back pain part of a general health pattern or is it a separate and distinctive entity? A critical literature review of comorbidity with low back pain. J Manipulative Physiol Ther 2003;26(4):243–252. 12750659 10.1016/s0161-4754(03)00003-4

[26] Schäfer I Kaduszkiewicz H Wagner H , et al. Reducing complexity: a visualisation of multimorbidity by combining disease clusters and triads BMC Public Health. 2014;14(1):1285. 25516155 10.1186/1471-2458-14-1285 PMC4301832

[27] Ashworth M Durbaba S Whitney D , et al. Journey to multimorbidity: longitudinal analysis exploring cardiovascular risk factors and sociodemographic determinants in an urban setting. BMJ Open 2019;9(12):e031649. 10.1136/bmjopen-2019-031649 PMC7008443 31874873

[28] Maher C Underwood M Buchbinder R . Non-specific low back pain. The Lancet 2017;389(10070):736–747. 10.1016/S0140-6736(16)30970-9 27745712

[29] Tran PB Kazibwe J Nikolaidis GF , et al. Costs of multimorbidity: a systematic review and meta-analyses. BMC Medicine 2022;20(1):234. 35850686 10.1186/s12916-022-02427-9 PMC9295506

[30] Stokes J Guthrie B Mercer SW , et al. Multimorbidity combinations, costs of hospital care and potentially preventable emergency admissions in England: A cohort study. PLoS Med 2021;18(1):e1003514. 33439870 10.1371/journal.pmed.1003514 PMC7815339

[31] Sund R . Quality of the Finnish Hospital Discharge Register: a systematic review. Scand J Public Health 2012;40(6):505–515. 22899561 10.1177/1403494812456637

[32] Ketola E Pitkänen V Huvinen S Seppälä T . Koko Suomen perusterveydenhuollon asiakaskirjo on nyt kuvattu. Suomen Lääkärilehti 2019:74:2027–2030.

[33] Matveinen U . Terveydenhuollon menot ja rahoitus 2019 - Terveydenhuollon menot kasvoivat kaikissa suurissa toiminnoissa. Tilastoraportti 15/2021. Terveyden ja hyvinvoinnin laitos 2021.

[34] Manssila S . Final report of the regional government, health and social services reform. Experiences of the preparatory work, lessons, and conclusions. Publications of the Ministry of Finance 2019:54.

[35] Finnish government . Programme of Prime Minister Sanna Marin’s Government 2019. Inclusive and competent Finland - a socially, economically and ecologically sustainable society. https://valtioneuvosto.fi/en/marin/government-programme (2022-12-02).

[36] Findata. Social and Health Data Permit Authority. Permits. https://findata.fi/en/permits/ (2023-09-08)

