# Supplementary material for: Multimorbidity transitions and the associated healthcare cost among the Finnish adult population during a two-year follow-up
Source: J Multimorb Comorb. 2023 Sep 12;13:26335565231202325. doi: 10.1177/26335565231202325 (PMC10498690; doi:10.1177/26335565231202325)
Supplement: Supplemental Material - Multimorbidity transitions and the associated healthcare cost among the Finnish adult population during a two-year follow-up [file sj-pdf-1-cob-10.1177_26335565231202325.pdf]

Supplementary Table 1. Disease groups and included diagnosis (ICD-10 and ICPC-2 codes)

**Disease group**	**ICD-10 codes**	**ICPC-2 codes**
1) Cancer and in-situ carcinomas	C00—C97, D00—D09	A79, B72—B74, D74—D77, L71, N74, R84, R85, S77, S79, T71, U75—U77, W72, X75—X77, Y77, Y78
2) Blood diseases and blood-forming organs	D50—D53, D55—D61, D63, D64, D66—D69, D71—89	B78—B84
3) Endocrine diseases	E00—E03, E05—E07, E20—E29, E31, E34, E35	T80, T85, T86
4) Diabetes	E10—E14	T89, T90
5) Obesity and other metabolic diseases	E65, E66, E68, E70—E72, E74—E80, E83—E85, E88—E90	T82, T93
6) Dementia and organic psychical disorders	F00—F09, G30—G32	P70, P71
7) Psychiatric and behavioural diseases related to substance abuse	F10—F16, F18, F19	P15, P18, P19
8) Schizophrenia and delusional diseases	F20—F22, F24, F25, F28, F29	P72
9) Mood disorders	F30—F39	P73, P76
10) Neurotic, stress-related and somatoform diseases, including eating disorders	F40—42, F44, F45, F48, F50, F52—F55, F59—F63, F68, F69	P02, P07, P74, P75, P78—P80, P86
11) Sleep disorders	F51, G47	P06
12) Other neurological diseases	G10—G13, G20—G26, G35, G37	N08, N86, N87
13) Epilepsy and migraine	G40—G44	N88—N90
14) Diseases of nerves and nerve-muscle junction	G50—G60, G62—G64, G70—G73	N91—N94
15) Chronic eye diseases and blindness	H17—H21, H25—H28, H31, H33, H35, H36, H40—H42, H47—H49, H51, H54	F82, F83, F85, F92—F95
16) Chronic ear diseases and deafness	H80—H83, H90, H91, H93, H95	H82, H83, H86
17) Hypertensive diseases	I10—I15, I95—I99	K86—K88
18) Ischemic heart diseases	I20—I25	K74—K76
19) Other diseases of the heart and pulmonary circulation	I27, I28, 131, I34—I39, I42—I45, I48—I51	K77—K80, K82—K84
20) Cerebrovascular diseases	I60—I69	K90, K91
21) Diseases or arteries and veins	I70—I73, I78, I79, I83—I87, I89	K92 , K96, K99, S97
22) Chronic diseases of the upper respiratory tract	J30—J35, J37, J38	R73, R75, R90, R97
23) Chronic diseases of the lower respiratory tract	J41—J47, J60—J68, J70, J84, J92, J95—J99	R79, R95, R96
24) Diseases of the oesophagus, stomach, and duodenum	K21—K31	D07, D84—D87
25) Inflammatory and other bowel diseases	K50—K52, K55, K57—K59, K62, K63, K90	D12, D92—D94
26) Diseases of the liver, pancreas, and biliary tract	K70—K77, K80, K81, K86	D97, D98
27) Chronic skin diseases	L10, L12, L13, L20, L21, L23, L24, L26, L28, L40, L41, L43—L45	S86, S87, S91
28) Inflammatory diseases of joints and connective tissue	M05—M14, M30—M36	L88, T92
29) Arthrosis	M15—M19	L89, L90, L91
30) Other musculoskeletal diseases	M20—M24, M70, M72—M75, M79—M84, M91, M93,M94, M96, M99	L92, L94, L95, L98
31) Back diseases	M40—M54	L01—L03, L83—L86
32) Chronic diseases of the kidneys and urinary tract	N01, N03—N05,N07, N08, N11, N18—N22, N31-N33, N35	U88, U95
33) Diseases of male genitals/reproductive organs	N40—N42, N48, N50	Y73, Y85
34) Diseases of female genitals/reproductive organs	N80, N81, N88	X87
